# Translating Basic Science Discoveries into Clinical Advances: Highlights from the EACR-AACR-IACR 2024 Conference in Celebration of Irish Association for Cancer Research’s 60th Anniversary

**DOI:** 10.3390/cancers17091420

**Published:** 2025-04-24

**Authors:** Seodhna M. Lynch, Cathy E. Richards, Aisling Ui Mhaonaigh, Niamh Lynam-Lennon, Alex J. Eustace, Emma H. Allott, Tracy Robson, Simone Marcone

**Affiliations:** 1 Personalised Medicine Centre, School of Medicine, Ulster University, C-TRIC Building, Altnagelvin Area Hospital, Glenshane Road, Londonderry BT47 6SB, UK; s.lynch1@ulster.ac.uk; 2 School of Dentistry, RCSI University of Medicine and Health Sciences, D02 YN77 Dublin, Ireland; 3 Department of Surgery, Trinity Translational Medicine Institute, Trinity St. James’s Cancer Institute, St. James’s Hospital, Trinity College Dublin, D08 NHY1 Dublin, Ireland; auimhaon@tcd.ie (A.U.M.); lynamlen@tcd.ie (N.L.-L.); 4 Department of Biology, Kathleen Lonsdale Institute for Human Health Research, Maynooth University, W23 NPY6 Maynooth, Ireland; 5 Life Science Institute, School of Biotechnology, Dublin City University, D09 NR58 Dublin, Ireland; alex.eustace@dcu.ie; 6 Patrick G. Johnston Centre for Cancer Research, Queen’s University Belfast, Belfast BT9 7AE, UK; e.allott@qub.ac.uk; 7 School of Pharmacy and Biomolecular Sciences, RCSI University of Medicine and Health Sciences, D02 YN77 Dublin, Ireland; 8 UCD School of Biology and Environmental Science, University College Dublin, D04 N2E5 Dublin, Ireland; simone.marcone1@ucd.ie

**Keywords:** Irish Association for Cancer Research (IACR), European Association for Cancer Research (EACR), American Association for Cancer Research (AACR), cancer research, immunotherapy, drug therapies, early cancer detection, tumour microenvironment, liquid biopsies, translational research, precision medicine

## Abstract

The EACR-AACR-IACR 2024 Basic and Translational Research Conference, held in Dublin, Ireland, from 27th–29th February, 2024, marked a significant milestone as part of the 60th anniversary celebrations of the Irish Association for Cancer Research (IACR). Organized in collaboration with the European Association for Cancer Research (EACR) and the American Association for Cancer Research (AACR), this prestigious event brought together leading experts in oncology research from around the world. The conference provided a platform for cutting-edge discussions on the latest advancements in immunotherapy, drug combinations, cell-based therapies, liquid biopsies, epigenetics, tumour microenvironment, and novel drug targets. With keynote lectures from esteemed researchers such as Kevan Shokat, Jerome Galon, Suzanne Topalian, and Scott Lowe, the conference facilitated knowledge exchange and fostered international collaboration in the pursuit of improved cancer treatments. The report highlights the key sessions, research breakthroughs, and discussions that shaped this landmark event.

## Simple Summary

The EACR-AACR-IACR 2024 Conference, held in Dublin, Ireland, marked the 60th anniversary of the Irish Association for Cancer Research (IACR). Organized in partnership with leading European and American cancer research organizations, the event brought together top scientists and clinicians to discuss the latest breakthroughs in cancer research. Over three days, experts explored new treatments, drug therapies, immunotherapy, and early cancer detection methods. Keynote speakers shared insights on improving cancer care. This report summarizes the most important topics discussed, highlighting progress in cancer research and future directions for better treatments.

## 1. Introduction

Cancer research is at the forefront of scientific discovery, continuously evolving with advancements in genomics, immunotherapy, and targeted drug development. The EACR-AACR-IACR 2024 Basic and Translational Research Conference, held in Dublin, Ireland, brought together leading cancer researchers, clinicians, and industry experts to discuss the latest breakthroughs in basic and translational oncology research. This conference was especially significant as it celebrated the 60th anniversary of the Irish Association for Cancer Research (IACR), highlighting its long-standing commitment to cancer research and innovation. The European Association for Cancer Research (EACR) and the American Association for Cancer Research (AACR) have a strong tradition of organizing Basic and Translational Research Conferences, and this event continued that legacy in partnership with the IACR. The conference provided a global platform for discussing recent advancements in immunology, drug development, tumour microenvironment, genomics, and epigenetics. By fostering collaboration among experts from diverse backgrounds, the event aimed to bridge the gap between laboratory discoveries and clinical applications, ensuring that new insights in cancer biology translate into improved patient care and therapeutic strategies. A key focus of the conference was bringing basic science discoveries to the clinic. Cancer is a complex disease driven by genetic mutations, epigenetic changes, and environmental factors, all of which influence tumour progression and treatment response. While advances in molecular and cellular research have led to significant progress in understanding cancer biology, the challenge remains in effectively translating these findings into clinical interventions. The scientific programme of the conference was designed to showcase cutting-edge research in cancer genomics, tumour microenvironment interactions, and immunotherapy. It explored new avenues for treating cancer through mechanism-based drug combinations, novel therapeutic targets, and personalized medicine approaches. In particular, recent discoveries have shed light on how conventional chemotherapy and immunotherapy elicit unexpected cellular responses, opening new opportunities for drug development and combination therapies. The conference was structured to appeal to a broad audience of scientists, clinicians, and translational researchers, ranging from experienced investigators to early-career trainees. Importantly, it also welcomed patients and patient advocates, fostering inclusive engagement across all stakeholders in the research process. Notably, this was the first time a Patient and Public Involvement (PPI) exhibitor stand was featured at the IACR conference, marking a significant step toward integrating patient perspectives into the scientific dialogue. Discussions were centred around key challenges in cancer research and treatment, with a focus on innovative strategies for tackling drug resistance, tumour heterogeneity, and the evolving landscape of immunotherapy. The programme covered a diverse range of topics, including immunotherapy, cell-based therapies, novel and undruggable targets, drug combinations, drug resistance, the precancer genome, liquid biopsies, preclinical models, the tumour microenvironment, and epigenetics. By bringing together experts from different disciplines, the EACR-AACR-IACR 2024 Conference served as an important forum for knowledge exchange, collaboration, and the advancement of cancer research. This report summarizes the major discussions and research highlights from the conference, providing key insights into the latest scientific developments and their potential impact on future cancer treatments.

## 2. Main Text

### 2.1. Opening Remarks

The conference was opened on 27 February 2024, by Professor René Bernards (Co-Chair EACR), Professor Christine M. Lovely (Co-Chair AACR), and Professor Tracy Robson (Co-Chair IACR). In the opening remarks, Professor Tracy Robson highlighted 2024 as the 60th anniversary of the IACR. Indeed, a dedicated IACR 60th Anniversary Special Symposium was held the day before this prestigious international meeting. Professor Robson highlighted the meaning underpinning the IACR logo. The logo was designed using the harp musical instrument, nucleobases, the Celtic knot, and the double helix to encapsulate how the IACR is All-Ireland focused and driven by cancer research and pivotal to this is building, nurturing, and strengthening connections between researchers, clinicians, and patients. Professor Robson welcomed all attendees, speakers, and sponsors to the meeting which highlighted the latest cutting-edge advances in the development of cancer and response to therapy from preclinical models to patients.

### 2.2. Special Session: IACR 60th Anniversary Special Symposium

A significant highlight of the conference was the discussion on advancing precision medicine through collaborative team science, emphasizing international cooperation in cancer research. The session commenced with a remote presentation by Prof. Henry Rodriguez on the National Cancer Institute’s CPTAC & ICPC initiatives (United States), which aim to accelerate precision medicine by integrating multiple approaches into cancer research. This was followed by a discussion on spatial single-cell analysis of stage II and III colorectal cancer, presented by Dr Fiona Ginty (GE HealthCare, United States), highlighting the significance of a US–Ireland–Northern Ireland Tripartite Collaboration that has contributed to understanding tumour heterogeneity and patient outcomes through advanced single-cell imaging technologies. The session continued with insights from Prof. Montserrat Garcia-Closas (The Institute of Cancer Research, UK) on the role of epidemiology and genetics consortia in advancing cancer risk assessment and personalized medicine, underscoring the value of large-scale collaborative networks in identifying genetic and environmental factors influencing cancer susceptibility. Further discussions led by Prof. Satish Gopal (Gillings School of Global Public Health, United States) and Prof. Mark Lawler (Queen’s University Belfast, Northen Ireland) centred on the Ireland–Northern Ireland–US NCI Cancer Consortium, emphasizing how cross-border research collaborations have strengthened cancer research and care, improving access to cutting-edge clinical trials and innovative treatments for patients across Ireland and Northern Ireland. The session continued with a focus on the All-Island Cancer Research Institute (AICRI), presented by Prof. William Gallagher (University College Dublin, Ireland). AICRI is a virtual institute that brings together the combined strengths of cancer researchers across the island of Ireland to create an overarching framework for cancer research. It encompasses a broad research program, from cancer prevention to diagnosis, treatment, and survivorship, aiming to develop more personalized treatment options and improve patient outcomes. AICRI has successfully united many academic institutions and multiple stakeholders from the healthcare sector, patient advocacy groups, and industry partners (https://www.aicri.org/). Prof. Mark Lawler discussed the critical importance of research in delivering 21st-century cancer care, presenting indicators from the Lancet Oncology European Groundshot Commission, which aims to address research inequalities and prioritize cancer care advancements across Europe. The session concluded with Prof. Catharine G. Young (US Cancer Moonshot, United States), who provided insights into the European Cancer Groundshot and US Cancer Moonshot initiatives, underscoring the global momentum in cancer research and the need for sustained funding, policy alignment, and international cooperation.

### 2.3. Opening Keynote Lecture: Professor Kevan Shokat

Professor Kevan Shokat from the University of California San Francisco, University of California Berkeley, and Howard Hughes Medical Institute, USA, delivered the opening keynote lecture titled “Overcoming the Undruggable Nature of the Most Common Human Oncogene K-Ras”. K-Ras, first discovered in 1983, is one of the most prevalent driver mutations in human cancer, yet no approved drugs specifically targeting K-Ras-driven tumours existed until 2021. This contrasts with B-Raf, discovered in 2002, which saw its first targeted drug approval by 2011. Professor Shokat’s research has focused on developing small molecule inhibitors capable of outcompeting K-Ras’s high affinity for GTP, a key challenge in drugging this oncogene [1]. Professor Shokat discussed multiple strategies for K-Ras inhibition, including blocking its membrane localization via farnesyl transferase (FTase) inhibitors (FTIs). While effective for H-Ras, this approach failed for K-Ras due to differences in mutational frequency and C-terminal sequences, allowing K-Ras to maintain its membrane localization despite FTase inhibition. This setback underscored the complexity of targeting K-Ras compared to other oncogenes. A breakthrough came when Professor Shokat’s team focused on the G12C mutation, a common K-Ras alteration that introduced a targetable amino acid. Using fragment-based drug discovery and disulfide tethering, his team screened for potential inhibitors binding to an allosteric pocket that was previously undetected—only accessible when the protein was in its inactive state [1]. This discovery paved the way for the development of sotorasib, the first FDA-approved KRAS-targeting drug for non-small cell lung cancer (NSCLC) [2]. Professor Shokat highlighted ongoing efforts to expand K-Ras inhibitors beyond G12C, emphasizing the need for new electrophilic compounds to target other K-Ras mutations [3]. However, resistance to G12C inhibitors is emerging, necessitating alternative strategies. He discussed bispecific T-cell engagers and radioligand therapy as promising approaches to enhance immune recognition of K-Ras mutant cancers, potentially overcoming resistance mechanisms [4,5,6]. His keynote lecture underscored the importance of targeting previously undruggable oncogenes and the future directions of K-Ras inhibitor development, offering new hope for patients with K-Ras-driven cancers.

### 2.4. Immunotherapy/Immuno-Oncology Plenary Session

The human immune system is often altered in ways that promote cancer growth; however, this system can also be harnessed to induce cancer regression. A significant focus of the EACR-AACR-IACR 2024 Basic and Translational Research Conference was the modulation of T-cell recognition in cancer treatment, an area of research led by Prof. Ton Schumacher at the Netherlands Cancer Institute. Prof. Schumacher’s work centres on understanding how T-cell responses can be leveraged for therapeutic benefit, particularly in the context of PD-1 blockade and neoantigen-specific T-cell recognition. A key innovation from Schumacher’s group is the development of a patient-derived tumour fragment (PDTF) platform, which enables the assessment of early immunological responses across various cancers. This system has revealed distinct patient groups: PDTF responders (PDTF-R) and non-responders (PDTF-NR) following anti-PD-1 therapy [7]. Notably, even among non-responders, some tumours exhibited brisk intratumoural CD8+ T-cell infiltration without corresponding pro-inflammatory cytokine secretion [7]. Further investigation demonstrated that PD-1 blockade increased INF-γ targets, particularly T-cell chemoattractant CXCL9 and CXCL10, which correlated with a reactivation of tumour-resident T-cells [7]. More recent studies from Schumacher’s team have highlighted CD8+ T-cell-derived IFN-γ as a key global modifier of the tumour microenvironment (TME), distinct from TNF-α, which acts locally [8]. Their research further revealed that T-cell pressure alters bystander tumour cells, shifting them toward IFN-γ-sensing transcriptional activity while reducing TGFβ-sensing, indicating a mutual negative regulation between these two cytokines [8]. These findings offer important insights into how the immune system can shape the tumour landscape and potentially improve immunotherapy strategies. Recognizing that each tumour presents a unique set of neoantigens and T-cell receptors (TCRs), Schumacher’s research aims to “crack the TCR-cancer recognition code”. His group is investigating the role of neoantigen-specific T-cells in cancer immunotherapy by examining how T-cells recognize mutated epitopes. A breakthrough in this area has been the development of a high-throughput genetic system, “HANSolo”, designed for the personalized identification of CD4+ and CD8+ T-cell-recognized antigens [9]. Additionally, their work has demonstrated a correlation between the presence of tumour-associated lymphoid structures (TLS) and intratumoural T-cell reactivation, positioning TLS as hotbeds for immune reactivation following PD-1 blockade [7]. Looking ahead, Schumacher’s group aims to integrate the PDTF platform with spatial profiling technologies to further elucidate the role of TLS in immune reactivation within human cancer tissues. They will also continue developing functional assays to refine the understanding of T-cell-driven tumour modification and immunotherapy response dynamics. Their ongoing research is expected to contribute significantly to the advancement of personalized cancer immunotherapy and precision medicine approaches.

### 2.5. Keynote Lecture: Dr Jerome Galon

Dr. Jérôme Galon, Director of Research at INSERM and Head of the Laboratory of Integrative Cancer Immunology, France, delivered a keynote lecture titled “From the Immune Contexture to Immunoscore: Basic to Clinic”. His presentation explored how tumour aggressiveness, progression, invasion, and recurrence are influenced by pre-existing immunity, which plays a key role in patient survival and response to immunotherapy. Dr. Galon introduced the concept of cancer immune contexture, which considers cell type, density, spatial distribution, and immune function within tumours [10]. Recognizing the need for a standardized immune-based classification system, he pioneered the development of the Immunoscore, introduced in 2020, to assess immune responses in cancer patients [11]. He also discussed metastasis and tumour evolution, emphasizing that current cancer evolution models (linear, neutral, big-bang, branched) focus only on tumour cells and fail to incorporate the role of the immune system. His work has led to a novel parallel immune selection model, describing how tumour cells and immune cells interact dynamically, driving immune escape and metastasis evolution [12]. Dr. Galon highlighted the future of using local immunity to guide therapy, particularly in rectal cancer [13]. The Immunoscore Biopsy (ISB) assay has been clinically validated to stratify rectal cancer patients into high-, intermediate-, and low-ISB groups, allowing for personalized treatment decisions. Patients with a high ISB score showed excellent outcomes, enabling some to avoid surgery and instead follow a watch-and-wait (WW) approach while achieving a complete response [14,15]. Transitioning to immunotherapy response prediction, he discussed the Immunoscore-IC (Immune Checkpoint) assay, which has been validated in non-small cell lung cancer (NSCLC) to predict response to anti-PD-L1 therapy and patient survival. A high Immunoscore-IC correlated with prolonged progression-free survival and overall survival [16]. Furthermore, in a phase 2 clinical trial (AtezoTRIBE) for metastatic colorectal cancer, a high Immunoscore-IC was associated with improved response to combination immunotherapy, demonstrating its predictive value in metastatic cancer treatment [17]. Dr. Galon also presented research on CAR-T therapy in diffuse large B-cell lymphoma (DLBCL), focusing on tumour immune microenvironment changes following CAR-T (Axi-cel) treatment. Data from the ZUMA-1 trial showed rapid and dramatic immune shifts associated with complete responses [18,19]. Additionally, findings from ZUMA-7, a second-line therapy trial for large B-cell lymphoma, revealed specific B-cell signatures predictive of better event-free survival in CAR-T-treated patients compared to standard therapies [20]. He concluded by emphasizing the role of the Immunosign-21 gene expression profile in guiding treatment choices and reinforcing the importance of assessing pre-existing immunity for personalized cancer therapies [21,22]. Dr. Galon’s work highlights the growing impact of immune-based classification systems, demonstrating how Immunoscore and Immunoscore-IC can guide clinical decision-making and enhance the effectiveness of cancer immunotherapy strategies.

### 2.6. The Precancer Genome Plenary Session

Somatic mutations occur continuously in healthy cells throughout a person’s lifetime, typically without leading to harmful consequences. However, in some cases, these mutations provide certain cells with a competitive advantage, allowing them to expand and potentially serve as the foundation for cancer initiation. A major focus of the conference was the earliest stages of cancer development, particularly how mutated stem cells gain the ability to spread through normal tissues. This research, led by Prof. Phil H. Jones and colleagues at the Wellcome Sanger Institute, UK, has been instrumental in uncovering the mechanisms driving clonal competition and somatic mutation accumulation in the skin and oesophagus. Prof. Jones’ team has examined somatic mutations and clonal selection in normal oesophageal tissue, identifying numerous positively selected clones that persist despite the absence of histological changes [23]. These findings suggest that early cancer development may begin long before observable tissue abnormalities emerge. Their studies have highlighted differences in mutations within the NOTCH1 and TP53 genes between normal and cancerous tissues, suggesting that these genes play a crucial role in modulating cancer risk [23]. Beyond genetic alterations, the group has discovered a novel tumour-protective mechanism in humans, distinct from immune surveillance, in which healthy cells with advantageous mutations actively outcompete and eliminate early cancerous growths [24]. This discovery opens potential new avenues for cancer prevention, as manipulating mutant cell populations in normal tissues may reduce the likelihood of early-stage cancers developing into malignancies [24]. A key area of investigation has been the disparity in NOTCH1 mutations between normal oesophageal epithelium and oesophageal tumours. Prof. Jones’ team has demonstrated how mutations in NOTCH1 confer a competitive advantage, allowing mutant clones to persist and expand within normal tissue [25]. Their findings suggest that mutations in one Notch1 allele enhance clonal expansion, and over time, this heterozygous population may continue to gain fitness advantages, leading to the loss of the remaining allele and full biallelic Notch1 mutations [25]. Given the high retention rate of at least one Notch1 allele in oesophageal squamous cell carcinoma (ESCC), their study indicates that wild-type Notch1 plays a tumour-suppressive role, warranting further investigation into the potential of anti-NOTCH1 inhibitors as therapeutic strategies for oesophageal neoplasms [25]. In addition to NOTCH1, the group has examined TP53 mutations across different stages of oesophageal carcinogenesis, elucidating how these mutations influence progenitor cell fate and clonal expansion [26]. Their work demonstrates that TP53 mutations drive biased progenitor cell proliferation, leading to the accumulation of mutant clones without significantly altering normal tissue architecture. However, while a single TP53 mutation alone presents a low risk of malignant transformation, the loss of the second allele permits additional genetic alterations, increasing genomic instability and promoting tumour formation [26]. Once established, p53 mutations further enhance tumour progression, making this pathway a crucial target for potential therapeutic interventions [26]. The group’s work to date highlights the importance of understanding clonal competition in epithelial carcinogenesis and the distinct effects of mutations at various transformation stages, as well as their potential therapeutic targeting.

### 2.7. Keynote Lecture: Professor Suzanne Topalian

Professor Suzanne Topalian from Johns Hopkins University (USA) delivered a keynote lecture titled “Neoadjuvant Immune Checkpoint Blockade: A Window of Opportunity to Advance Cancer Immunotherapy” [27]. Her presentation focused on how co-regulatory pathways modulate tumour–immune cell interactions and how immune checkpoint blockade (ICB) can enhance anti-tumour immune responses in the neoadjuvant setting. A central theme of her talk was the PD-1/PD-L1 pathway, a critical target in cancer immunotherapy. Blocking PD-1 or PD-L1 can lead to tumour regression in cases where other therapies have failed [28]. Professor Topalian highlighted that the FDA has approved nine different PD-1/PD-L1 monoclonal antibodies for use in 23 different types of advanced cancers, establishing anti-PD-(L)1 therapy as a foundational treatment approach [29]. However, she emphasized that pancreatic and prostate cancers remain largely unresponsive to anti-PD-(L)1 monotherapy, highlighting the need for alternative or combination strategies in these malignancies. Predicting patient response to anti-PD-(L)1 therapy remains a challenge. While the FDA has approved three biomarkers—PD-L1 immunohistochemistry, microsatellite instability (MSI), and tumour mutational burden (TMB)—these are not universally effective across all tumour types. To address this, ongoing research is developing multimodal biomarker panels using machine learning. In melanoma studies conducted at Johns Hopkins, these multimodal panels outperformed any single biomarker in predicting treatment response [30]. Traditionally, immune checkpoint blockade has been used in the adjuvant setting, but Professor Topalian postulated that these therapies should be prioritized in the neoadjuvant setting. The main objective of neoadjuvant PD-1 blockade is to prime a systemic anti-tumour immune response and prevent relapse by targeting micro-metastases before surgery [27]. This approach offers several advantages, including enhancing T-cell priming, increasing available tumour tissue for biomarker research, and enabling more personalized therapy strategies. Previously, biomarker analysis was restricted to limited needle biopsy samples, but neoadjuvant checkpoint blockade allows for larger tissue samples from surgical resections, facilitating in-depth immunological studies. Professor Topalian highlighted the role of RNA sequencing (RNAseq) in Merkel cell carcinoma (MCC), which has distinguished responders from non-responders to therapy. Additionally, single-cell RNAseq studies in non-small cell lung cancer (NSCLC) identified multiple upregulated molecules in non-responders, offering potential targets for new combination therapies [31]. Professor Topalian concluded by emphasizing the transformative potential of neoadjuvant immune checkpoint blockade, highlighting its role in priming systemic immunity, preventing relapse, and predicting treatment outcomes. She noted that large tumour samples from neoadjuvant-treated patients will enhance correlative studies, improving the understanding of resistance mechanisms and optimizing future immunotherapy strategies.

### 2.8. Liquid Biopsies Plenary Session

Extracellular vesicles (EVs) are emerging as a key component of liquid biopsies, offering significant potential in cancer diagnostics, treatment monitoring, and understanding tumour biology. In her presentation, Prof. Lorraine O’Driscoll from Trinity College Dublin (Ireland) emphasized the critical role of EVs in intercellular communication and their potential as biomarkers in liquid biopsies [32]. These nanosized vesicles, released by both normal and cancerous cells, carry a diverse array of biomolecules, including proteins, lipids, RNA, and DNA, which reflect the physiological and pathological state of their cells of origin. One of the key advantages of EVs in liquid biopsies is their ability to encapsulate and protect their molecular cargo from enzymatic degradation, preserving crucial information about the tumour microenvironment [33,34]. Prof. O’Driscoll highlighted how EVs play a fundamental role in cancer progression by facilitating metastasis, immune modulation, and therapy resistance [35,36]. Tumour-derived EVs can travel through bodily fluids such as blood, urine, and saliva, making them accessible targets for non-invasive cancer detection. Recent advancements in isolation and characterization techniques, such as ultracentrifugation, size-exclusion chromatography, and immunoaffinity-based approaches, have improved the ability to analyse EVs with greater specificity and sensitivity [37,38]. A major focus of the presentation was the role of EVs in therapy resistance. Prof. O’Driscoll’s research has demonstrated that EVs can transfer drug resistance-associated molecules, including specific proteins and microRNAs [35,39]. By profiling EV-associated biomarkers, clinicians may be able to tailor treatment strategies in real time, improving patient outcomes. Prof. O’Driscoll’s talk also highlighted the potential of EVs as prognostic and predictive biomarkers in cancer response to treatment [34,40]. Since EVs reflect the molecular characteristics of the tumour, their detection in patients post-treatment can indicate the presence of residual cancer cells before clinical symptoms arise or before they are detectable through imaging techniques. This makes EVs particularly valuable in personalized medicine, where early intervention can significantly alter disease progression.

One of the key components of liquid biopsies discussed by Prof. O’Driscoll was the detection and characterization of circulating tumour cells. These rare cells, shed from primary and metastatic tumours into the bloodstream, offer crucial information on cancer progression and therapeutic resistance [41,42]. Another vital biomarker analysed through liquid biopsies is circulating tumour DNA (ctDNA), which consists of fragmented DNA released by tumour cells. The analysis of ctDNA enables the detection of specific genetic mutations, epigenetic changes, and clonal evolution, aiding in treatment selection and the identification of emerging resistance mechanisms [43,44]. Despite their promise, challenges remain in standardizing EV isolation and analysis techniques to ensure reproducibility and clinical translation. Prof. O’Driscoll underscored the need for further research and collaboration in refining EV-based liquid biopsies, optimizing their clinical application, and integrating them into routine cancer care. As research progresses, the growing understanding of EV biology and function is expected to unlock new opportunities for precision oncology, ultimately enhancing early detection, treatment monitoring, and therapeutic decision-making.

### 2.9. Tumour Microenvironment Plenary Session

The tumour microenvironment (TME) is the complex ecosystem surrounding a tumour, consisting of immune cells, stromal cells, blood vessels, and signalling molecules. It plays a critical role in tumour growth, metastasis, and response to therapy, often creating a supportive niche for cancer survival. Understanding and targeting the TME is key to developing new cancer treatments that can overcome resistance and enhance immune responses [45]. Professor Johanna Joyce from the University of Lausanne (Switzerland) presented her research on therapeutically exploiting the TME. Her work focuses on how interactions between cancer, immune, and stromal cells influence tumour progression, metastasis, and therapy response, particularly in brain cancer, where two-thirds of cases are metastatic and patient prognosis remains poor. Brain metastases are an understudied area, affecting 10–40% of cancer patients during disease progression. Using 350 patient samples, her team identified neutrophil infiltration in brain metastases, particularly in the perivascular region, altering the neutrophil-to-lymphocyte ratio (NLR). RNA sequencing (RNAseq) revealed that blood and brain neutrophils differ, with brain-infiltrating neutrophils enriched in inflammatory pathways. These tumour-associated neutrophils (TANs) exhibit increased longevity, modulated by tumour-conditioned media (TCM), which downregulates apoptotic genes [46]. Notably, this neutrophil alteration was not tumour-derived but induced by interactions with blood vessels, mediated by TNFα and ceruloplasmin in the brain TME [46]. Professor Joyce also discussed the role of brain vasculature in immune modulation, showing that genes linked to angiogenesis and interferon signalling are upregulated, with implications for therapy [47]. Her team identified CD276 as a key immune checkpoint regulator in brain metastases. In preclinical models, anti-CD276 antibody treatment significantly reduced tumour volume, improved survival, and strengthened the blood–brain barrier, decreasing fibrinogen expression while increasing I-CAM and V-CAM, which are critical for immune cell infiltration [47]. Her findings suggest that targeting CD276 and inflammatory mediators could be an effective strategy for treating brain metastases. Developing TME-targeted therapies for brain malignancies could help counteract tumour-supporting microenvironments, instead leveraging them to enhance anti-tumour responses.

### 2.10. Epigenetics Plenary Session

Epigenetic dysregulation plays a key role in cancer initiation and progression, altering gene expression patterns by silencing tumour suppressor genes and activating oncogenes. A crucial player in this process is the Polycomb Repressive Complex 2 (PRC2), which modifies chromatin structures through histone methylation, specifically H3K27 trimethylation (H3K27me3) [48]. Studies have shown that PRC2 disruption contributes to various cancer types, highlighting its significance as a potential therapeutic target [49]. Professor Adrian Bracken from Trinity College Dublin (Ireland) presented his research on cancer epigenetics, focusing on PRC2 regulation in cell-fate decisions and tumour development. His group has identified vertebrate-specific polycombs PALI1 and PALI2, which bind to PRC2 and enhance its methyltransferase activity [50]. These proteins may link polycombs with transcriptional co-repressors, influencing cellular identity regulation in both development and cancer. His team also demonstrated that accessory proteins of PRC2.1 and PRC2.2 subcomplexes play essential roles in recruiting PRC2 to chromatin and regulating H3K27me3 deposition [48]. Their work further contextualized the evolutionary conservation of PRC2.1 and PRC2.2, revealing distinct functions for each subcomplex [51]. Notably, their research validated that JARID2, a PRC2.2 component, drives CBX7-cPRC1 recruitment, introducing a new mechanism governing Polycomb system function [51]. A significant part of Professor Bracken’s work focuses on diffuse midline gliomas (DMG), where 80% of cases carry mutations in histone H3 genes, particularly H3F3A, leading to the K27M mutation (H3.3-K27M) [52]. His research demonstrated that H3.3-K27M is an early driver mutation in gliomas, which retains PRC2 at specific chromatin regions, influencing tumour development [52]. Using an isogenic model, his team also showed that PRC2 inhibition can counteract some pathogenic effects of the H3.3-K27M mutation, suggesting new therapeutic opportunities for targeting PRC2 in gliomas [52]. Professor Bracken’s ongoing research aims to further explore H3K27me3 landscapes in cancer, paving the way for novel epigenetic therapies that could disrupt tumour-promoting chromatin modifications and offer new treatment avenues for aggressive malignancies.

### 2.11. Closing Keynote Lecture: Professor Scott Lowe

Professor Scott Lowe from the Howard Hughes Medical Institute, Sloan Kettering Institute, and Memorial Sloan Kettering Cancer Centre, USA, delivered the closing keynote lecture titled “Harnessing Senescence Biology for Cancer Suppression”. His presentation explored the dual nature of senescence, highlighting both its tumour-suppressive and pro-tumorigenic effects. Senescence, first identified by Leonard Hayflick in the 1960s, involves stable cell cycle arrest and a secretory program known as the senescence-associated secretory phenotype (SASP). While senescence can suppress tumorigenesis and aid tissue homeostasis, it can also promote inflammation and contribute to age-related tissue decline. Professor Lowe examined how mutant K-Ras and injury trigger a progenitor-like state in pancreatic cancer, with p53 acting as a barrier to malignant transformation. Cells that lose p53 persist, allowing pre-cancerous states to evolve into invasive cancer, demonstrating p53’s role in preventing neoplastic reprogramming and plasticity [53,54,55]. He also discussed targeting cell surface proteins like urokinase plasminogen activator receptor (uPAR) in senescence. uPAR, which plays a role in wound healing, exists in a soluble circulating form (suPAR) that serves as a biomarker of disease severity [56,57]. His team has shown that uPAR-positive cells accumulate with age and display senescent features, making them a target for senolytic therapies. A novel approach using uPAR CAR T cells selectively eliminates senescent cells, demonstrating efficacy against liver fibrosis with minimal toxicity [56,57]. Professor Lowe presented findings that uPAR CAR T cells persist in aged mice, reduce aging features, and show strong anti-tumour activity in human ovarian cancer xenografts and patient-derived xenografts (PDXs). Importantly, human uPAR CAR T cells exhibit sustained efficacy against tumour rechallenge and appear safe in a humanised immune system. His concluding message emphasized the clinical potential of senolytic CAR T cells for treating uPAR-positive cancers in fibrotic environments and other senescence-associated diseases.

## 3. Discussion

The EACR-AACR-IACR 2024 Conference was a landmark event, not only for its contributions to cancer research but also as a celebration of the 60th anniversary of the IACR. This milestone underscored the long-standing commitment of IACR to fostering scientific excellence and international collaboration in oncology. Throughout the conference, speakers highlighted the progress made over the past decades in cancer diagnostics, treatment, and translational research, emphasizing the importance of continued innovation and global partnerships in the fight against cancer. This conference also represented a significant step toward greater inclusivity and patient engagement within the research community. For the first time, a dedicated Patient and Public Involvement (PPI) exhibitor stand was featured at IACR, underscoring a growing recognition of the value of integrating patient perspectives into scientific discourse. The presence of patients and patient advocates, alongside scientists, clinicians, and translational researchers at various career stages, enriched the dialogue and highlighted the importance of collaborative approaches in driving meaningful research outcomes. The primary goal of this conference report is to summarize and communicate the main scientific highlights and emerging themes presented during the sessions. Given the format, it does not provide detailed critical evaluation or in-depth discussion of individual studies. However, several presentations throughout the conference highlighted important translational challenges, such as overcoming drug resistance, improving biomarker validation, and integrating novel diagnostics and therapeutics into existing clinical frameworks. We believe that acknowledging these practical aspects adds further context to the scientific advances discussed and may prompt continued dialogue and collaboration across the research and clinical communities.

A key theme of the conference was the role of multidisciplinary collaboration in advancing precision medicine. The IACR 60th Anniversary Special Symposium brought together leading researchers from Ireland, the United States, and Europe to discuss the future of cancer research and the impact of international cooperation. Cross-border initiatives such as the Ireland–Northern Ireland–US NCI Cancer Consortium and the All-Island Cancer Research Institute (AICRI) showcased how collaborative research frameworks have significantly improved cancer care, facilitated cutting-edge clinical trials, and strengthened infrastructure for translational research. Among the major breakthroughs discussed was the development of novel therapeutic strategies targeting previously undruggable oncogenes. Professor Kevan Shokat’s keynote lecture on K-Ras inhibition provided a compelling narrative on how small molecule inhibitors are overcoming historical challenges in targeting one of the most prevalent oncogenes in cancer. The successful development of sotorasib and ongoing efforts to expand targeted therapy beyond the G12C mutation reflect a transformative shift in cancer drug development. These advancements, along with new findings in tumour microenvironment (TME) research, highlight the increasing recognition of immune and stromal interactions in driving tumour progression and therapy resistance. The work presented on immune checkpoint regulators such as CD276 demonstrated the potential of targeting the TME to enhance therapeutic efficacy. Immunotherapy remained a central focus of the conference, with significant discussions on predictive biomarkers, immune profiling, and neoadjuvant checkpoint blockade strategies. Professor Jerome Galon’s presentation on Immunoscore reinforced the value of immune-based classifications in refining patient stratification for immunotherapy. His parallel immune selection model provided new insights into the mechanisms of tumour metastasis and immune escape, reshaping current perspectives on cancer evolution. Similarly, Professor Suzanne Topalian’s keynote lecture on neoadjuvant immune checkpoint blockade emphasized how early intervention with immunotherapy can enhance systemic anti-tumour immune responses, improve surgical outcomes, and prevent relapse. The incorporation of immune profiling tools such as RNA sequencing and single-cell analyses was highlighted as a critical step toward personalizing immunotherapy approaches. A particularly cutting-edge session focused on extracellular vesicles (EVs) and liquid biopsies, which are emerging as key tools for non-invasive cancer diagnostics. Professor Lorraine O’Driscoll’s research on EV-mediated drug resistance provided compelling evidence that EVs facilitate therapy resistance by transferring drug resistance-associated proteins and microRNAs between tumour cells. The ability to profile EV-associated biomarkers in real time presents an opportunity to refine treatment strategies and monitor disease progression more effectively. Additionally, the role of liquid biopsies in detecting minimal residual disease (MRD) and early relapse was explored, emphasizing their potential in guiding clinical decisions and improving long-term patient outcomes. The conference’s focus on epigenetics further demonstrated how chromatin modifications and histone methylation contribute to cancer progression and therapy resistance. Professor Adrian Bracken’s research on PRC2 regulation in diffuse midline gliomas (DMGs) provided new insights into the role of H3K27M mutations in driving glioma genesis. His findings underscored the importance of targeting PRC2 dysfunction in developing novel epigenetic therapies for aggressive malignancies. This session reinforced the growing interest in epigenetic regulators as therapeutic targets, opening new avenues for personalized cancer treatment. One of the most innovative discussions of the conference centred on harnessing senescence biology for cancer suppression. Professor Scott Lowe’s closing keynote lecture presented groundbreaking research on senolytic CAR T cells targeting uPAR, a novel approach to selectively eliminating senescent tumour cells. His findings demonstrated the feasibility of using CAR T-cell therapy to counteract tumour-promoting senescence, a strategy with far-reaching implications not only in oncology but also in aging-related diseases. Overall, the EACR-AACR-IACR 2024 Conference provided a comprehensive overview of the latest advancements in cancer research, from immunotherapy and tumour microenvironment interactions to liquid biopsies, epigenetics, and drug resistance mechanisms. Importantly, the celebration of IACR’s 60th anniversary served as a reminder of the progress achieved over the past six decades and the critical role that international collaboration, innovation, and scientific rigor play in shaping the future of cancer care. While challenges remain in translating these discoveries into routine clinical practice, the conference reinforced a collective commitment to bridging the gap between basic science and clinical applications. Future research efforts should continue to focus on biomarker validation, overcoming therapy resistance, and optimizing combination treatment strategies. By fostering interdisciplinary partnerships and integrating cutting-edge technologies, the future of cancer treatment promises more effective, personalized, and accessible solutions for patients worldwide.

## 4. Conclusions

The EACR-AACR-IACR 2024 Conference celebrated 60 years of IACR, highlighting key advancements in precision oncology, immunotherapy, and liquid biopsies. A major theme was bridging research and clinical practice, ensuring breakthroughs reach patients faster. To achieve this, biomarker validation, clinical trial optimization, and regulatory support must be strengthened. Collaboration between researchers, clinicians, and industry is crucial for translating discoveries into effective treatments. By integrating real-time patient data, expanding access to personalized therapies, and fostering global partnerships, cancer care can become more precise, accessible, and impactful for all patients.

## Data Availability

The data presented in this study are available in this article.

## Abbreviations

AACR	American Association for Cancer Research
AICRI	All-Island Cancer Research Institute
ctDNA	Circulating Tumour DNA
CTCs	Circulating Tumour Cells
CXCL9/CXCL10	Chemokines involved in immune cell recruitment
DMG	Diffuse Midline Glioma
EACR	European Association for Cancer Research
EVs	Extracellular Vesicles
FTIs	Farnesyl Transferase Inhibitors
GTP	Guanosine Triphosphate
H3K27me3	Histone H3 Lysine 27 Trimethylation
IACR	Irish Association for Cancer Research
ICB	Immune Checkpoint Blockade
ICPC	International Cancer Proteogenome Consortium
INF-γ	Interferon Gamma
ISB	Immunoscore Biopsy
KRAS	Kirsten Rat Sarcoma Viral Oncogene
MCC	Merkel Cell Carcinoma
MRD	Minimal Residual Disease
MSI	Microsatellite Instability
NCI	National Cancer Institute
NSCLC	Non-Small Cell Lung Cancer
PDTF	Patient-Derived Tumour Fragment
PRC2	Polycomb Repressive Complex 2
RNAseq	RNA Sequencing
SASP	Senescence-Associated Secretory Phenotype
TLS	Tumour-Associated Lymphoid Structures
TME	Tumour Microenvironment
TMB	Tumour Mutational Burden
TNF-α	Tumour Necrosis Factor Alpha
uPAR	Urokinase Plasminogen Activator Receptor
WW	Watch-and-Wait Approach

## References

[1] Ostrem J.M., Peters U., Sos M.L., Wells J.A., Shokat K.M. (2013). K-Ras(G12C) inhibitors allosterically control GTP affinity and effector interactions. Nature.

[2] Skoulidis F., Li B.T., Dy G.K., Price T.J., Falchook G.S., Wolf J., Italiano A., Schuler M., Borghaei H., Barlesi F. (2021). Sotorasib for Lung Cancers with KRAS p.G12C Mutation. N. Engl. J. Med..

[3] Zhang Z., Morstein J., Ecker A.K., Guiley K.Z., Shokat K.M. (2022). Chemoselective Covalent Modification of K-Ras(G12R) with a Small Molecule Electrophile. J. Am. Chem. Soc..

[4] Zhang Z., Rohweder P.J., Ongpipattanakul C., Basu K., Bohn M.F., Dugan E.J., Steri V., Hann B., Shokat K.M., Craik C.S. (2022). A covalent inhibitor of K-Ras(G12C) induces MHC class I presentation of haptenated peptide neoepitopes targetable by immunotherapy. Cancer Cell.

[5] Hattori T., Maso L., Araki K.Y., Koide A., Hayman J., Akkapeddi P., Bang I., Neel B.G., Koide S. (2023). Creating MHC-Restricted Neoantigens with Covalent Inhibitors That Can Be Targeted by Immune Therapy. Cancer Discov..

[6] Awad M.M., Liu S., Rybkin I.I., Arbour K.C., Dilly J., Zhu V.W., Johnson M.L., Heist R.S., Patil T., Riely G.J. (2021). Acquired Resistance to KRAS(G12C) Inhibition in Cancer. N. Engl. J. Med..

[7] Voabil P., de Bruijn M., Roelofsen L.M., Hendriks S.H., Brokamp S., Braber M.V.D., Broeks A., Sanders J., Herzig P., Zippelius A. (2021). An ex vivo tumor fragment platform to dissect response to PD-1 blockade in cancer. Nat. Med..

[8] Hoekstra M.E., Slagter M., Urbanus J., Toebes M., Slingerland N., de Rink I., Kluin R.J., Nieuwland M., Kerkhoven R., Wessels L.F. (2024). Distinct spatiotemporal dynamics of CD8+ T cell-derived cytokines in the tumor microenvironment. Cancer Cell.

[9] Cattaneo C.M., Battaglia T., Urbanus J., Moravec Z., Voogd R., de Groot R., Hartemink K.J., Haanen J.B.A.G., Voest E.E., Schumacher T.N. (2023). Identification of patient-specific CD4+ and CD8+ T cell neoantigens through HLA-unbiased genetic screens. Nat. Biotechnol..

[10] Galon J., Costes A., Sanchez-Cabo F., Kirilovsky A., Mlecnik B., Lagorce-Pagès C., Tosolini M., Camus M., Berger A., Wind P. (2006). Type, density, and Location of Immune Cells Within Human Colorectal Tumors Predict Clinical Outcome. Science.

[11] Angell H.K., Bruni D., Barrett J.C., Herbst R., Galon J. (2020). The Immunoscore: Colon Cancer and Beyond. Clin. Cancer Res..

[12] Angelova M., Mlecnik B., Vasaturo A., Bindea G., Fredriksen T., Lafontaine L., Buttard B., Morgand E., Bruni D., Jouret-Mourin A. (2018). Evolution of Metastases in Space and Time under Immune Selection. Cell.

[13] Galon J., Bruni D. (2019). Approaches to treat immune hot, altered and cold tumours with combination immunotherapies. Nat. Rev. Drug Discov..

[14] El Sissy C., Kirilovsky A., Pagès C.L., Marliot F., Custers P.A., Dizdarevic E., Sroussi M., Castillo-Martin M., Haicheur N., Dermani M. (2024). International Validation of the Immunoscore Biopsy in Patients With Rectal Cancer Managed by a Watch-and-Wait Strategy. J. Clin. Oncol..

[15] Kirilovsky A., El Sissy C., Zeitoun G., Marliot F., Haicheur N., Lagorce-Pagès C., Taieb J., Karoui M., Custers P., Dizdarevic E. (2022). The “Immunoscore” in rectal cancer: Could we search quality beyond quantity of life?. Oncotarget.

[16] Ghiringhelli F., Bibeau F., Greillier L., Fumet J.D., Ilie A., Monville F., Lauge C., Catteau A., Boquet I., Majdi A. (2023). Immunoscore immune checkpoint using spatial quantitative analysis of CD8 and PD-L1 markers is predictive of the efficacy of anti- PD1/PD-L1 immunotherapy in non-small cell lung cancer. eBioMedicine.

[17] Antoniotti C., Rossini D., Pietrantonio F., Catteau A., Salvatore L., Lonardi S., Boquet I., Tamberi S., Marmorino F., Moretto R. (2022). Upfront FOLFOXIRI plus bevacizumab with or without atezolizumab in the treatment of patients with metastatic colorectal cancer (AtezoTRIBE): A multicentre, open-label, randomised, controlled, phase 2 trial. Lancet Oncol..

[18] Scholler N., Perbost R., Locke F.L., Jain M.D., Turcan S., Danan C., Chang E.C., Neelapu S.S., Miklos D.B., Jacobson C.A. (2022). Tumor immune contexture is a determinant of anti-CD19 CAR T cell efficacy in large B cell lymphoma. Nat. Med..

[19] Locke F.L., Ghobadi A., Jacobson C.A., Miklos D.B., Lekakis L.J., Oluwole O.O., Lin Y., Braunschweig I., Hill B.T., Timmerman J.M. (2019). Long-term safety and activity of axicabtagene ciloleucel in refractory large B-cell lymphoma (ZUMA-1): A single-arm, multicentre, phase 1-2 trial. Lancet Oncol..

[20] Locke F.L., Miklos D.B., Jacobson C.A., Perales M.-A., Kersten M.-J., Oluwole O.O., Ghobadi A., Rapoport A.P., McGuirk J., Pagel J.M. (2022). Axicabtagene Ciloleucel as Second-Line Therapy for Large B-Cell Lymphoma. N. Engl. J. Med..

[21] Bruni D., Angell H.K., Galon J. (2020). The immune contexture and Immunoscore in cancer prognosis and therapeutic efficacy. Nat. Rev. Cancer.

[22] Locke F.L., Filosto S., Chou J., Vardhanabhuti S., Perbost R., Dreger P., Hill B.T., Lee C., Zinzani P.L., Kröger N. (2024). Impact of tumor microenvironment on efficacy of anti-CD19 CAR T cell therapy or chemotherapy and transplant in large B cell lymphoma. Nat. Med..

[23] Martincorena I., Fowler J.C., Wabik A., Lawson A.R.J., Abascal F., Hall M.W.J., Cagan A., Murai K., Mahbubani K., Stratton M.R. (2018). Somatic mutant clones colonize the human esophagus with age. Science.

[24] Colom B., Herms A., Hall M.W.J., Dentro S.C., King C., Sood R.K., Alcolea M.P., Piedrafita G., Fernandez-Antoran D., Ong S.H. (2021). Mutant clones in normal epithelium outcompete and eliminate emerging tumours. Nature.

[25] Abby E., Dentro S.C., Hall M.W.J., Fowler J.C., Ong S.H., Sood R., Herms A., Piedrafita G., Abnizova I., Siebel C.W. (2023). Notch1 mutations drive clonal expansion in normal esophageal epithelium but impair tumor growth. Nat. Genet..

[26] Murai K., Dentro S., Ong S.H., Sood R., Fernandez-Antoran D., Herms A., Kostiou V., Abnizova I., Hall B.A., Gerstung M. (2022). p53 mutation in normal esophagus promotes multiple stages of carcinogenesis but is constrained by clonal competition. Nat. Commun..

[27] Topalian S.L., Forde P.M., Emens L.A., Yarchoan M., Smith K.N., Pardoll D.M. (2023). Neoadjuvant immune checkpoint blockade: A window of opportunity to advance cancer immunotherapy. Cancer Cell.

[28] Zou W., Wolchok J.D., Chen L. (2016). PD-L1 (B7-H1) and PD-1 pathway blockade for cancer therapy: Mechanisms, response biomarkers, and combinations. Sci. Transl. Med..

[29] Topalian S.L., Drake C.G., Pardoll D.M. (2015). Immune checkpoint blockade: A common denominator approach to cancer therapy. Cancer Cell.

[30] Anagnostou V., Bruhm D.C., Niknafs N., White J.R., Shao X.M., Sidhom J.W., Stein J., Tsai H.-L., Wang H., Belcaid Z. (2020). Integrative Tumor and Immune Cell Multi-omic Analyses Predict Response to Immune Checkpoint Blockade in Melanoma. Cell Rep. Med..

[31] Caushi J.X., Zhang J., Ji Z., Vaghasia A., Zhang B., Hsiue E.H., Mog B.J., Hou W., Justesen S., Blosser R. (2021). Transcriptional programs of neoantigen-specific TIL in anti-PD-1-treated lung cancers. Nature.

[32] Soekmadji C., Li B., Huang Y., Wang H., An T., Liu C., Pan W., Chen J., Cheung L., Falcon-Perez J.M. (2020). The future of Extracellular Vesicles as Theranostics—An ISEV meeting report. J. Extracell. Vesicles.

[33] Useckaite Z., Mukhopadhya A., Moran B., O'Driscoll L. (2020). Extracellular vesicles report on the MET status of their cells of origin regardless of the method used for their isolation. Sci. Rep..

[34] Daly R., O'Driscoll L. (2021). Extracellular vesicles in blood: Are they viable as diagnostic and predictive tools in breast cancer?. Drug Discov. Today.

[35] O’brien K., Lowry M.C., Corcoran C., Martinez V.G., Daly M., Rani S., Gallagher W.M., Radomski M.W., MacLeod R.A., O’driscoll L. (2015). miR-134 in extracellular vesicles reduces triple-negative breast cancer aggression and increases drug sensitivity. Oncotarget.

[36] Martinez V.G., O'Neill S., Salimu J., Breslin S., Clayton A., Crown J., O’Driscoll L. (2017). Resistance to HER2-targeted anti-cancer drugs is associated with immune evasion in cancer cells and their derived extracellular vesicles. OncoImmunology.

[37] Mukhopadhya A., Santoro J., Moran B., Useckaite Z., O’Driscoll L. (2021). Optimisation and comparison of orthogonal methods for separation and characterisation of extracellular vesicles to investigate how representative infant milk formula is of milk. Food Chem..

[38] Martinez-Pacheco S., O’driscoll L. (2021). Evidence for the Need to Evaluate More Than One Source of Extracellular Vesicles, Rather Than Single or Pooled Samples Only, When Comparing Extracellular Vesicles Separation Methods. Cancers.

[39] Namee N.M., O’Driscoll L. (2018). Extracellular vesicles and anti-cancer drug resistance. Biochim. Biophys. Acta Rev. Cancer.

[40] Corcoran C., Rani S., O’Driscoll L. (2014). miR-34a is an intracellular and exosomal predictive biomarker for response to docetaxel with clinical relevance to prostate cancer progression. Prostate.

[41] Lewis F., Beirne J., Henderson B., Norris L., Cadoo K., Kelly T., Martin C., Hurley S., Kanjuga M., O’Driscoll L. (2024). Unravelling the biological and clinical challenges of circulating tumour cells in epithelial ovarian carcinoma. Cancer Lett..

[42] Friel A.M., Corcoran C., Crown J., O'Driscoll L. (2010). Relevance of circulating tumor cells, extracellular nucleic acids, and exosomes in breast cancer. Breast Cancer Res. Treat.

[43] Li P., Liu S., Du L., Mohseni G., Zhang Y., Wang C. (2022). Liquid biopsies based on DNA methylation as biomarkers for the detection and prognosis of lung cancer. Clin. Epigenetics.

[44] Constâncio V., Nunes S.P., Henrique R., Jerónimo C. (2020). DNA Methylation-Based Testing in Liquid Biopsies as Detection and Prognostic Biomarkers for the Four Major Cancer Types. Cells.

[45] Klemm F., Maas R.R., Bowman R.L., Kornete M., Soukup K., Nassiri S., Brouland J.P., Iacobuzio-Donahue C.A., Brennan C., Tabar V. (2020). Interrogation of the Microenvironmental Landscape in Brain Tumors Reveals Disease-Specific Alterations of Immune Cells. Cell.

[46] Maas R.R., Soukup K., Fournier N., Massara M., Galland S., Kornete M., Wischnewski V., Lourenco J., Croci D., Alvarez-Prado A.F. (2023). The local microenvironment drives activation of neutrophils in human brain tumors. Cell.

[47] Bejarano L., Kauzlaric A., Lamprou E., Lourenco J., Fournier N., Ballabio M., Colotti R., Maas R., Galland S., Massara M. (2024). Interrogation of endothelial and mural cells in brain metastasis reveals key immune-regulatory mechanisms. Cancer Cell.

[48] Healy E., Mucha M., Glancy E., Fitzpatrick D.J., Conway E., Neikes H.K., Monger C., Van Mierlo G., Baltissen M.P., Koseki Y. (2019). PRC2.1 and PRC2.2 Synergize to Coordinate H3K27 Trimethylation. Mol. Cell.

[49] Conway E., Healy E., Bracken A.P. (2015). PRC2 mediated H3K27 methylations in cellular identity and cancer. Curr. Opin. Cell Biol..

[50] Conway E., Jerman E., Healy E., Ito S., Holoch D., Oliviero G., Deevy O., Glancy E., Fitzpatrick D.J., Mucha M. (2018). A Family of Vertebrate-Specific Polycombs Encoded by the LCOR/LCORL Genes Balance PRC2 Subtype Activities. Mol. Cell.

[51] Glancy E., Wang C., Tuck E., Healy E., Amato S., Neikes H.K., Mariani A., Mucha M., Vermeulen M., Pasini D. (2023). PRC2.1- and PRC2.2-specific accessory proteins drive recruitment of different forms of canonical PRC1. Mol. Cell.

[52] Brien G.L., Bressan R.B., Monger C., Gannon D., Lagan E., Doherty A.M., Healy E., Neikes H., Fitzpatrick D.J., Deevy O. (2021). Simultaneous disruption of PRC2 and enhancer function underlies histone H3.3-K27M oncogenic activity in human hindbrain neural stem cells. Nat. Genet..

[53] Tschaharganeh D.F., Xue W., Calvisi D.F., Evert M., Michurina T.V., Dow L.E., Banito A., Katz S.F., Kastenhuber E.R., Weissmueller S. (2014). p53-dependent Nestin regulation links tumor suppression to cellular plasticity in liver cancer. Cell.

[54] Burdziak C., Alonso-Curbelo D., Walle T., Reyes J., Barriga F.M., Haviv D., Xie Y., Zhao Z., Zhao C.J., Chen H.-A. (2023). Epigenetic plasticity cooperates with cell-cell interactions to direct pancreatic tumorigenesis. Science.

[55] Alonso-Curbelo D., Ho Y.-J., Burdziak C., Maag J.L.V., Morris J.P., Chandwani R., Chen H.-A., Tsanov K.M., Barriga F.M., Luan W. (2021). A gene–environment-induced epigenetic program initiates tumorigenesis. Nature.

[56] Amor C., Fernández-Maestre I., Chowdhury S., Ho Y.-J., Nadella S., Graham C., Carrasco S.E., Nnuji-John E., Feucht J., Hinterleitner C. (2024). Prophylactic and long-lasting efficacy of senolytic CAR T cells against age-related metabolic dysfunction. Nat. Aging.

[57] Amor C., Feucht J., Leibold J., Ho Y.-J., Zhu C., Alonso-Curbelo D., Mansilla-Soto J., Boyer J.A., Li X., Giavridis T. (2020). Senolytic CAR T cells reverse senescence-associated pathologies. Nature.

